# 4-(1-Naphth­yl)benzoic acid

**DOI:** 10.1107/S1600536809046339

**Published:** 2009-11-07

**Authors:** Carlos F. R. A. C. Lima, Ligia R. Gomes, Luís M. N. B. F. Santos, John Nicolson Low

**Affiliations:** a Centro de Investigação em Química, Departamento de Química, Faculdade de Ciências, Universidade do Porto, Rua do Campo Alegre 687, P-4169_007 Porto, Portugal; b REQUIMTE, Departamento de Química, Faculdade de Ciências, Universidade do Porto, Rua do Campo Alegre 687, P-4169_007 Porto, Portugal; c Department of Chemistry, University of Aberdeen, Meston Walk, Old Aberdeen AB24 3UE, Scotland

## Abstract

In the title mol­ecule, C_17_H_12_O_2_, the dihedral angle between the mean plane of the benzene ring and that of the naphthalene ring system is 49.09 (6)°. In the crystal structure, mol­ecules are linked to form centrosymmetric dimers *via* inter­molecular O—H⋯O hydrogen bonds. The hydr­oxy H atom is disordered over two sites with refined occupancies of 0.62 (3) and 0.38 (3).

## Related literature

For a description of supra­molecular structures formed *via* hydrogen bonds, see: Bernstein *et al.* (1995 ▶).
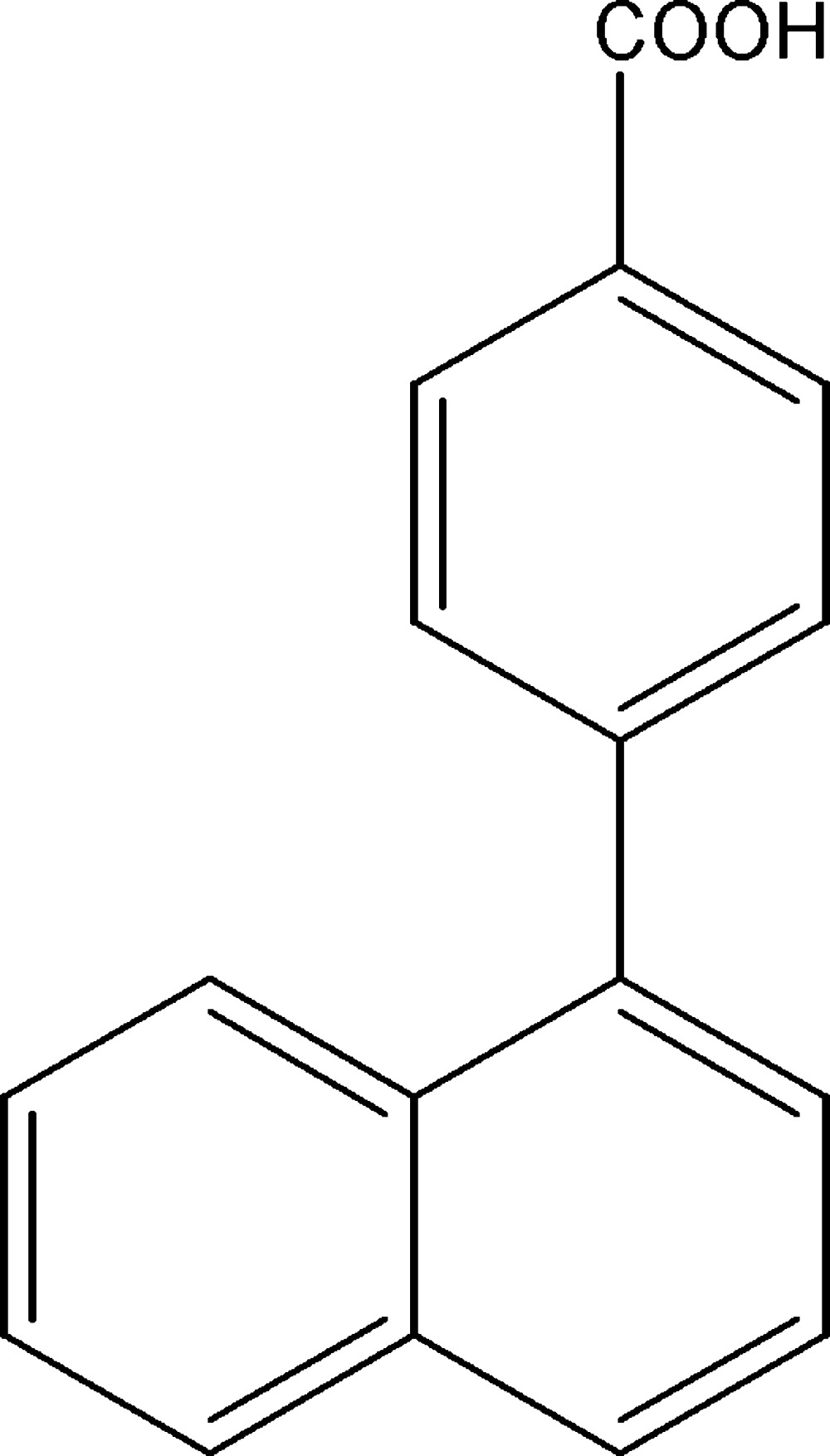


## Experimental

### 

#### Crystal data

C_17_H_12_O_2_
*M* *_r_* = 248.27
Monoclinic, 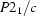

*a* = 3.8972 (6) Å
*b* = 40.511 (6) Å
*c* = 7.6106 (12) Å
β = 99.323 (3)°
*V* = 1185.7 (3) Å^3^
*Z* = 4
Mo *K*α radiation
μ = 0.09 mm^−1^
*T* = 150 K
0.30 × 0.18 × 0.02 mm

#### Data collection

Bruker SMART APEXII diffractometer
Absorption correction: multi-scan (*SADABS*; Bruker, 2003 ▶) *T*_min_ = 0.973, *T*_max_ = 0.998
4700 measured reflections
2412 independent reflections
1954 reflections with *I* > 2σ(*I*)
*R*_int_ = 0.023

#### Refinement

*R*[*F* ^2^ > 2σ(*F* ^2^)] = 0.044
*wR*(*F* ^2^) = 0.107
*S* = 1.04
2412 reflections
174 parameters
H-atom parameters constrained
Δρ_max_ = 0.25 e Å^−3^
Δρ_min_ = −0.17 e Å^−3^

### 

Data collection: *APEX2* (Bruker, 2004 ▶); cell refinement: *APEX2* and *SAINT* (Bruker, 2004 ▶); data reduction: *SAINT*; program(s) used to solve structure: *SHELXS97* (Sheldrick, 2008 ▶); program(s) used to refine structure: *SHELXL97* (Sheldrick, 2008 ▶); molecular graphics: *ORTEPII* (Johnson, 1976 ▶) and *PLATON* (Spek, 2009 ▶); software used to prepare material for publication: *SHELXL97*.

## Supplementary Material

Crystal structure: contains datablocks global, I. DOI: 10.1107/S1600536809046339/lh2945sup1.cif

Structure factors: contains datablocks I. DOI: 10.1107/S1600536809046339/lh2945Isup2.hkl

Additional supplementary materials:  crystallographic information; 3D view; checkCIF report

## Figures and Tables

**Table 1:** Hydrogen-bond geometry (Å, °)

*D*—H⋯*A*	*D*—H	H⋯*A*	*D*⋯*A*	*D*—H⋯*A*
O41—H41⋯O42^i^	0.84	1.79	2.6161 (18)	170
O42—H42⋯O41^i^	0.88	1.75	2.6161 (18)	168

Symmetry code: (i) 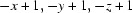.

**Fig. 1.**
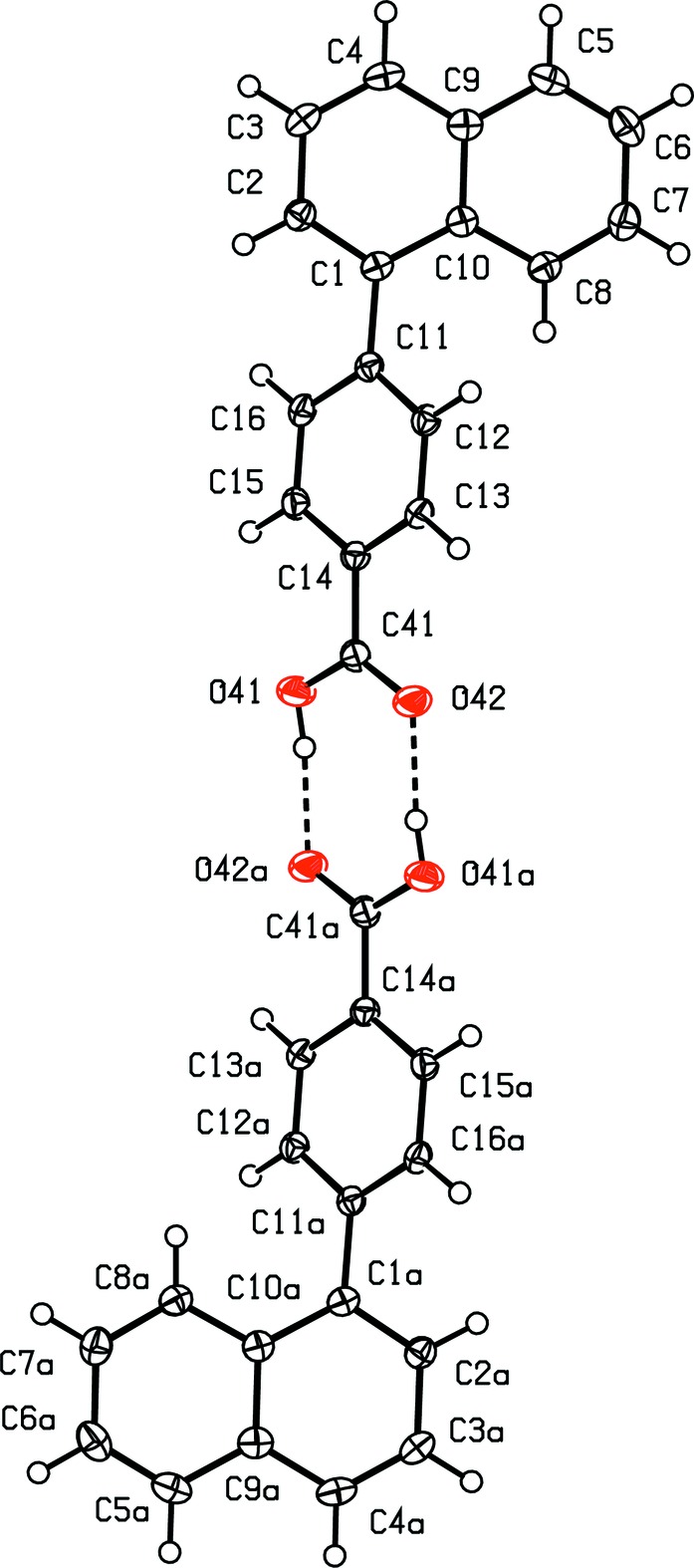
A centrosymmetric dimer of the title compound. Atoms labelled with an 'a' are related by the symmetry operator (1 - x,1 - y,1 - z). Displacement ellipsoids are drawn at the 30% probability level. Only the major component of the disorder is shown.

**Fig. 2.**
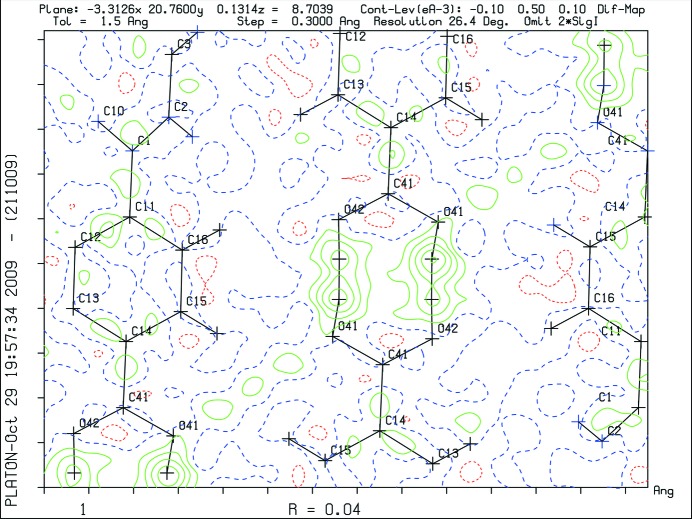
A difference map with hydroxy H atoms not included in the structure model, showing a section in the plane of the disordered hydroxy H atoms and the C atom of the carboxyl group.

## supplementary crystallographic information

### Comment

In the crystal structure, molecules of the title compound form typical carboxylic acid *R*^2^_2_(8), (Bernstein *et al.* 1995), dimers across inversion centers. The hydroxy H atom is disordered over two sites. Figure 1 shows a centrosymmetric dimer of the title compound.

### Experimental

A solution of K_2_CO_3_(20 mmol,4 mol/eq) in 20 ml of water was added to a solution of 1-bromonaphthalene(5 mmol, 1 mol/eq), 4-carboxyphenylboronic acid (8 mmol of water, 1.6 mol/eq) and Pd(OAc)_2_ (2mol%) in 20 ml of water. The resultant mixture was heated at 95°C, with constant stirring, for 6 h. The final solution was allowed to cool to room temperature, acidified to pH < 5 and extracted with ethyl acetate. The organic layer was washed with aqueous 0.1MHCl, dried over anhydrous sodium sulfate and evaporated. The resulting precipitate was washed with ether yielding 0.73 g of white flakes, (yield 59%, purity 99.9%). Crystals suitable for X-ray diffraction were obtained by crystallization from a 50/50 mixture of chloroform and acetone.

### Refinement

H atoms positions were calulated and refined as riding atoms with C—H(aromatic), 0.95 Å. The *O*—*H*(hydroxy) was located in a difference Fourier map and identified as disordered over two sites, one H atom attached to O41 with a distance of 0.84Å and a site occupancy of 0.62 (3), the other attached to O42 with a distance of 0.88Å and a site occupancy of 0.38 (3). These atoms were refined as riding atoms. These positions were confirmed by examination of a difference map with hydroxy H atoms omitted form the structure model after the final refinement cycle (see Fig 2). The reflections 020 and 040 were omitted from the refinement since they were obscured by the beam-stop. The asymmetric unit was selected so that the centre of the dimer lies at (1/2, 1/2, 1/2).

### Crystal data

**Table:**

C_17_H_12_O_2_	*F*(000) = 520
*M**_r_* = 248.27	*D*_x_ = 1.391 Mg m^−^^3^
Monoclinic, *P*2_1_/*c*	Melting point: 509 K
Hall symbol: -P 2ybc	Mo *K*α radiation, λ = 0.71073 Å
*a* = 3.8972 (6) Å	Cell parameters from 1563 reflections
*b* = 40.511 (6) Å	θ = 6.3–26.4°
*c* = 7.6106 (12) Å	µ = 0.09 mm^−^^1^
β = 99.323 (3)°	*T* = 150 K
*V* = 1185.7 (3) Å^3^	Plate, colorless
*Z* = 4	0.30 × 0.18 × 0.02 mm

### Data collection

**Table:**

Bruker SMART APEXII diffractometer	2412 independent reflections
Radiation source: fine-focus sealed tube	1954 reflections with *I* > 2σ(*I*)
graphite	*R*_int_ = 0.023
Detector resolution: 8.333 pixels mm^-1^	θ_max_ = 26.4°, θ_min_ = 2.9°
ω scans	*h* = −2→4
Absorption correction: multi-scan (*SADABS*; Bruker, 2003)	*k* = −45→50
*T*_min_ = 0.973, *T*_max_ = 0.998	*l* = −9→9
4700 measured reflections	

### Refinement

**Table:**

Refinement on *F*^2^	Secondary atom site location: difference Fourier map
Least-squares matrix: full	Hydrogen site location: inferred from neighbouring sites
*R*[*F*^2^ > 2σ(*F*^2^)] = 0.044	H-atom parameters constrained
*wR*(*F*^2^) = 0.107	*w* = 1/[σ^2^(*F*_o_^2^) + (0.0453*P*)^2^ + 0.4999*P*] where *P* = (*F*_o_^2^ + 2*F*_c_^2^)/3
*S* = 1.04	(Δ/σ)_max_ = 0.001
2412 reflections	Δρ_max_ = 0.25 e Å^−^^3^
174 parameters	Δρ_min_ = −0.17 e Å^−^^3^
0 restraints	Extinction correction: *SHELXL97* (Sheldrick, 2008), Fc^*^=kFc[1+0.001xFc^2^λ^3^/sin(2θ)]^-1/4^
Primary atom site location: structure-invariant direct methods	Extinction coefficient: 0.008 (2)

### Special details

**Table:**

Geometry. All e.s.d.'s (except the e.s.d. in the dihedral angle between two l.s. planes) are estimated using the full covariance matrix. The cell e.s.d.'s are taken into account individually in the estimation of e.s.d.'s in distances, angles and torsion angles; correlations between e.s.d.'s in cell parameters are only used when they are defined by crystal symmetry. An approximate (isotropic) treatment of cell e.s.d.'s is used for estimating e.s.d.'s involving l.s. planes.
Refinement. Refinement of *F*^2^ against ALL reflections. The weighted *R*-factor *wR* and goodness of fit *S* are based on *F*^2^, conventional *R*-factors *R* are based on *F*, with *F* set to zero for negative *F*^2^. The threshold expression of *F*^2^ > σ(*F*^2^) is used only for calculating *R*-factors(gt) *etc*. and is not relevant to the choice of reflections for refinement. *R*-factors based on *F*^2^ are statistically about twice as large as those based on *F*, and *R*- factors based on ALL data will be even larger.

### Fractional atomic coordinates and isotropic or equivalent isotropic displacement parameters (Å^2^)

**Table:**

	*x*	*y*	*z*	*U*_iso_*/*U*_eq_	Occ. (<1)
O41	0.5693 (4)	0.50335 (3)	0.73150 (17)	0.0320 (3)	
H41	0.4832	0.4923	0.6419	0.048*	0.62 (3)
O42	0.7354 (4)	0.53531 (3)	0.52203 (16)	0.0330 (3)	
H42	0.6502	0.5202	0.4440	0.050*	0.38 (3)
C1	1.1807 (4)	0.62769 (4)	1.2149 (2)	0.0187 (4)	
C2	1.3777 (4)	0.61873 (4)	1.3748 (2)	0.0214 (4)	
H2	1.4358	0.5961	1.3961	0.026*	
C3	1.4950 (4)	0.64219 (5)	1.5074 (2)	0.0248 (4)	
H3	1.6296	0.6353	1.6166	0.030*	
C4	1.4159 (5)	0.67478 (5)	1.4795 (2)	0.0260 (4)	
H4	1.5015	0.6905	1.5683	0.031*	
C5	1.1179 (5)	0.71913 (4)	1.2917 (2)	0.0266 (4)	
H5	1.2060	0.7349	1.3797	0.032*	
C6	0.9075 (5)	0.72919 (4)	1.1408 (2)	0.0288 (4)	
H6	0.8491	0.7519	1.1242	0.035*	
C7	0.7765 (5)	0.70599 (4)	1.0094 (2)	0.0267 (4)	
H7	0.6281	0.7131	0.9047	0.032*	
C8	0.8613 (4)	0.67326 (4)	1.0313 (2)	0.0224 (4)	
H8	0.7689	0.6579	0.9414	0.027*	
C9	1.2078 (4)	0.68535 (4)	1.3196 (2)	0.0216 (4)	
C10	1.0846 (4)	0.66168 (4)	1.1855 (2)	0.0190 (4)	
C11	1.0745 (4)	0.60175 (4)	1.0782 (2)	0.0180 (4)	
C12	1.1205 (4)	0.60601 (4)	0.9008 (2)	0.0197 (4)	
H12	1.2256	0.6257	0.8670	0.024*	
C13	1.0158 (4)	0.58211 (4)	0.7744 (2)	0.0196 (4)	
H13	1.0497	0.5854	0.6548	0.024*	
C14	0.8601 (4)	0.55312 (4)	0.8215 (2)	0.0192 (4)	
C15	0.8267 (4)	0.54788 (4)	0.9991 (2)	0.0209 (4)	
H15	0.7294	0.5278	1.0334	0.025*	
C16	0.9353 (4)	0.57188 (4)	1.1253 (2)	0.0203 (4)	
H16	0.9147	0.5680	1.2462	0.024*	
C41	0.7169 (4)	0.52934 (4)	0.6815 (2)	0.0215 (4)	

### Atomic displacement parameters (Å^2^)

**Table:**

	*U*^11^	*U*^22^	*U*^33^	*U*^12^	*U*^13^	*U*^23^
O41	0.0478 (8)	0.0241 (7)	0.0241 (7)	−0.0120 (6)	0.0064 (6)	−0.0021 (5)
O42	0.0525 (9)	0.0286 (7)	0.0179 (6)	−0.0113 (6)	0.0056 (6)	−0.0031 (5)
C1	0.0165 (8)	0.0231 (9)	0.0172 (8)	−0.0026 (7)	0.0047 (6)	−0.0008 (6)
C2	0.0207 (8)	0.0243 (9)	0.0193 (8)	0.0012 (7)	0.0042 (6)	0.0003 (7)
C3	0.0238 (9)	0.0331 (10)	0.0166 (8)	−0.0004 (8)	0.0008 (6)	−0.0005 (7)
C4	0.0260 (9)	0.0323 (10)	0.0196 (9)	−0.0054 (8)	0.0035 (7)	−0.0075 (7)
C5	0.0301 (10)	0.0227 (9)	0.0290 (9)	−0.0060 (8)	0.0110 (8)	−0.0065 (7)
C6	0.0341 (10)	0.0208 (9)	0.0350 (10)	0.0031 (8)	0.0156 (8)	0.0023 (8)
C7	0.0274 (10)	0.0290 (10)	0.0242 (9)	0.0041 (8)	0.0060 (7)	0.0045 (7)
C8	0.0225 (9)	0.0249 (9)	0.0199 (8)	−0.0024 (7)	0.0034 (6)	−0.0008 (7)
C9	0.0197 (9)	0.0247 (9)	0.0217 (8)	−0.0033 (7)	0.0072 (7)	−0.0021 (7)
C10	0.0171 (8)	0.0218 (9)	0.0193 (8)	−0.0024 (7)	0.0064 (6)	0.0006 (6)
C11	0.0147 (8)	0.0198 (8)	0.0189 (8)	0.0030 (6)	0.0014 (6)	−0.0001 (6)
C12	0.0190 (8)	0.0206 (9)	0.0193 (8)	−0.0013 (7)	0.0023 (6)	0.0025 (7)
C13	0.0203 (8)	0.0220 (9)	0.0165 (8)	0.0012 (7)	0.0027 (6)	0.0016 (6)
C14	0.0186 (8)	0.0194 (8)	0.0189 (8)	0.0019 (7)	0.0011 (6)	−0.0008 (6)
C15	0.0226 (9)	0.0188 (9)	0.0211 (8)	0.0000 (7)	0.0028 (6)	0.0034 (7)
C16	0.0221 (9)	0.0226 (9)	0.0163 (8)	0.0019 (7)	0.0035 (6)	0.0035 (6)
C41	0.0231 (9)	0.0200 (9)	0.0217 (8)	0.0018 (7)	0.0042 (6)	0.0014 (7)

### Geometric parameters (Å, °)

**Table:**

O41—C41	1.286 (2)	C6—H6	0.9500
O41—H41	0.8400	C7—C8	1.370 (2)
O42—C41	1.251 (2)	C7—H7	0.9500
O42—H42	0.8806	C8—C10	1.423 (2)
C1—C2	1.379 (2)	C8—H8	0.9500
C1—C10	1.435 (2)	C9—C10	1.426 (2)
C1—C11	1.489 (2)	C11—C16	1.396 (2)
C2—C3	1.407 (2)	C11—C12	1.401 (2)
C2—H2	0.9500	C12—C13	1.379 (2)
C3—C4	1.365 (3)	C12—H12	0.9500
C3—H3	0.9500	C13—C14	1.395 (2)
C4—C9	1.415 (2)	C13—H13	0.9500
C4—H4	0.9500	C14—C15	1.395 (2)
C5—C6	1.361 (3)	C14—C41	1.477 (2)
C5—C9	1.420 (2)	C15—C16	1.384 (2)
C5—H5	0.9500	C15—H15	0.9500
C6—C7	1.407 (3)	C16—H16	0.9500
			
C41—O41—H41	109.6	C4—C9—C10	119.45 (16)
C41—O42—H42	116.4	C5—C9—C10	119.66 (16)
C2—C1—C10	118.95 (15)	C8—C10—C9	117.32 (15)
C2—C1—C11	118.87 (15)	C8—C10—C1	123.64 (15)
C10—C1—C11	122.17 (14)	C9—C10—C1	119.00 (15)
C1—C2—C3	121.67 (16)	C16—C11—C12	118.02 (15)
C1—C2—H2	119.2	C16—C11—C1	120.51 (14)
C3—C2—H2	119.2	C12—C11—C1	121.44 (14)
C4—C3—C2	120.18 (16)	C13—C12—C11	121.06 (15)
C4—C3—H3	119.9	C13—C12—H12	119.5
C2—C3—H3	119.9	C11—C12—H12	119.5
C3—C4—C9	120.68 (16)	C12—C13—C14	120.24 (15)
C3—C4—H4	119.7	C12—C13—H13	119.9
C9—C4—H4	119.7	C14—C13—H13	119.9
C6—C5—C9	121.00 (17)	C13—C14—C15	119.33 (15)
C6—C5—H5	119.5	C13—C14—C41	119.53 (14)
C9—C5—H5	119.5	C15—C14—C41	121.06 (15)
C5—C6—C7	119.97 (17)	C16—C15—C14	119.96 (15)
C5—C6—H6	120.0	C16—C15—H15	120.0
C7—C6—H6	120.0	C14—C15—H15	120.0
C8—C7—C6	120.50 (17)	C15—C16—C11	121.24 (14)
C8—C7—H7	119.8	C15—C16—H16	119.4
C6—C7—H7	119.8	C11—C16—H16	119.4
C7—C8—C10	121.51 (16)	O42—C41—O41	122.96 (15)
C7—C8—H8	119.2	O42—C41—C14	119.94 (15)
C10—C8—H8	119.2	O41—C41—C14	117.08 (14)
C4—C9—C5	120.89 (16)		
			
C10—C1—C2—C3	−1.9 (2)	C11—C1—C10—C9	−177.44 (14)
C11—C1—C2—C3	178.11 (14)	C2—C1—C11—C16	46.9 (2)
C1—C2—C3—C4	−0.3 (3)	C10—C1—C11—C16	−133.14 (16)
C2—C3—C4—C9	1.7 (3)	C2—C1—C11—C12	−131.12 (17)
C9—C5—C6—C7	−0.2 (3)	C10—C1—C11—C12	48.9 (2)
C5—C6—C7—C8	−0.5 (3)	C16—C11—C12—C13	3.1 (2)
C6—C7—C8—C10	−0.5 (3)	C1—C11—C12—C13	−178.82 (15)
C3—C4—C9—C5	178.49 (16)	C11—C12—C13—C14	0.2 (2)
C3—C4—C9—C10	−1.0 (2)	C12—C13—C14—C15	−3.0 (2)
C6—C5—C9—C4	−177.62 (16)	C12—C13—C14—C41	173.74 (15)
C6—C5—C9—C10	1.9 (2)	C13—C14—C15—C16	2.5 (2)
C7—C8—C10—C9	2.1 (2)	C41—C14—C15—C16	−174.24 (15)
C7—C8—C10—C1	179.89 (15)	C14—C15—C16—C11	0.9 (2)
C4—C9—C10—C8	176.77 (15)	C12—C11—C16—C15	−3.7 (2)
C5—C9—C10—C8	−2.7 (2)	C1—C11—C16—C15	178.25 (15)
C4—C9—C10—C1	−1.2 (2)	C13—C14—C41—O42	−0.1 (2)
C5—C9—C10—C1	179.34 (15)	C15—C14—C41—O42	176.61 (17)
C2—C1—C10—C8	−175.23 (15)	C13—C14—C41—O41	−178.49 (15)
C11—C1—C10—C8	4.8 (2)	C15—C14—C41—O41	−1.8 (2)
C2—C1—C10—C9	2.6 (2)		

### Hydrogen-bond geometry (Å, °)

**Table:**

*D*—H···*A*	*D*—H	H···*A*	*D*···*A*	*D*—H···*A*
O41—H41···O42^i^	0.84	1.79	2.6161 (18)	170
O42—H42···O41^i^	0.88	1.75	2.6161 (18)	168

Symmetry codes: (i) −*x*+1, −*y*+1, −*z*+1.
